# Childhood and maternal infections and risk of acute leukaemia in children with Down syndrome: a report from the Children's Oncology Group

**DOI:** 10.1038/sj.bjc.6602223

**Published:** 2004-11-02

**Authors:** K N Canfield, L G Spector, L L Robison, D Lazovich, M Roesler, A F Olshan, F O Smith, N A Heerema, D R Barnard, C K Blair, J A Ross

**Affiliations:** 1 Division of Epidemiology, University of Minnesota School of Public Health, Minneapolis, MN 55454, USA; 2 Department of Pediatrics, University of Minnesota Medical School, Minneapolis, MN 55455, USA; 3 University of Minnesota Cancer Center, USA; 4 Department of Epidemiology, University of North Carolina, Chapel Hill, NC 27599, USA; 5 Cincinnati Children's Hospital Medical Center, Cincinnati, OH 45229, USA; 6 Department of Pathology, The Ohio State University, Columbus, OH 43210, USA; 7 IWK Health Centre, Halifax, NS B3J 3G9, USA

**Keywords:** leukaemia, Down syndrome, infections

## Abstract

Children with Down syndrome (DS) are highly susceptible to acute leukaemia. Given the potential role of infections in the aetiology of leukaemia in children without DS, we investigated whether there was an association between early-life infections and acute leukaemia in children with DS. Maternal infections during pregnancy were also examined. We enrolled 158 incident cases of acute leukaemia in children with DS (97 acute lymphoblastic leukaemia (ALL) and 61 acute myeloid leukaemia (AML)) diagnosed at Children's Oncology Group institutions between 1997 and 2002. DS controls (*N*=173) were selected from the cases' primary care clinics and frequency matched on age at leukaemia diagnosis. Data were collected on demographics, child's medical history, mother's medical history, and other factors by maternal interview. Analyses were conducted using unconditional logistic regression adjusted for potential confounders. A significant negative association was observed between acute leukaemia and any infection in the first 2 years of life (adjusted odds ratio (OR)=0.55, 95% confidence interval (CI) (0.33–0.92); OR=0.53, 95% CI (0.29–0.97); and OR=0.59, 95% CI (0.28–1.25) for acute leukaemia combined, ALL, and AML respectively). The association between acute leukaemia and maternal infections during pregnancy was in the same direction but not significant. This study offers support for the hypothesis that early-life infections may play a protective role in the aetiology of acute leukaemia in children with DS.

While the aetiology of childhood leukaemia remains largely unknown (Ross *et al*, 1994; Pui, 1995), the association of childhood acute leukaemia with Down syndrome (DS) has been recognised for more than 70 years (Brewster and Cannon, 1930). Children with DS, the most common chromosomal abnormality in the United States (Centers for Disease Control & Prevention, 1994) have an estimated 10- to 30-fold increased risk of developing acute leukaemia compared to children without DS (Fong and Brodeur, 1987; Robison, 1992; Taub, 2001).

In addition to being the most common genetic disorder linked to the development of leukaemia, trisomy 21 is one of the most common acquired abnormalities in the leukaemic cells of patients without DS (Sakurai and Swansbury, 1984). Several genes on chromosome 21 may be disrupted in children with acute leukaemia and specific gene translocations involving chromosome 21 have been implicated in leukaemia development (Okuda *et al*, 1998). These findings represent accumulating evidence that genes on chromosome 21 may be causally linked to the development of leukaemia. Despite this evidence, it is estimated that only 1% of children with DS will ever develop the disease (Taub, 2001). Host genetic susceptibility and environmental exposures likely work together to give rise to leukaemia (Greaves, 1988). For children with DS, trisomy 21 may confer increased genetic susceptibility and represent the first step in a complex multistep pathway to leukemogenesis (Scholl *et al*, 1982; Ross, 1999). Additional environmental exposures, including those thought to be related to leukaemia in children without DS, are probably also required. Thus, investigations of environmental risk factors for leukaemia in children with DS may help untangle complex gene–environment interactions and provide clues not only to the aetiology of acute leukaemia in children with DS but also for leukaemia in the general population.

Greaves proposed that childhood acute lymphoblastic leukaemia (ALL), a variant comprising the majority of childhood leukaemia, develops as the result of at least two independent and sequential genetic mutations (Greaves *et al*, 1985; Greaves, 1988). The first mutation occurs during the expansion of B-cell precursors *in utero*, giving rise to a population of preleukaemic clone cells (Greaves, 1988; Greaves *et al*, 2003); the second mutation occurs in a mutant clone during postnatal proliferation of B cells. Greaves hypothesised that exposure to common infections in early childhood may protect the child against ALL by contributing to normal maturation of the immune system, whereas children whose exposure is delayed will be at comparatively higher risk (Greaves, 1988, 1997; Greaves and Alexander, 1993). Some studies have supported the possible role of delayed infection in the aetiology of childhood leukaemia (van Steensel-Moll *et al*, 1986; McKinney *et al*, 1999; Schuz *et al*, 1999; Neglia *et al*, 2000). However, results have been inconsistent, potentially due to the heterogeneity of childhood leukaemia and insufficient study power to test the hypothesis within leukaemia subgroups (Ross, 1999; Jourdan-Da Silva *et al*, 2004).

We investigated whether there was an association between infections and acute leukaemia among children with DS. Specifically, we evaluated whether children with DS and leukaemia experienced more infections in the first 2 years of life than did children with DS who did not have leukaemia. In addition, we examined whether case mothers experienced more urinary tract infections (UTIs) and kidney infections during pregnancy than did control mothers.

## METHODS

### Case identification

Children with DS diagnosed with incident acute leukaemia between January 1997 and October 2002 were identified through the registration files of the Children's Oncology Group (COG). COG formed from the merger of the Children's Cancer Group and the Paediatric Oncology Group and consists of 238 member institutions in the United States, Canada, Australia, and Europe. Together they care for an estimated 94% of leukaemia patients under the age of 15 years and 73% of leukaemia patients under the age of 20 years in the United States (Ross *et al*, 1996; Liu *et al*, 2003).

The eligibility criteria for cases were: a diagnosis of incident acute leukaemia at age 19 years or earlier, residence in the United States or Canada at the time of diagnosis, presence of a telephone in the home (for comparability to a second group of control children without DS who were selected through random digit dialing), and the availability of an English-speaking biological mother for interview. In addition, authorisation from the patient's COG physician to contact the child's parents, and written consent for interview from the mother were required. Institutional Review Boards of the University of Minnesota and participating COG institutions approved this study.

### Control identification

Upon completion of the telephone interview, case mothers were asked to provide the name and address of the physician responsible for their child's primary care before diagnosis of leukaemia. These physicians were contacted and asked to provide a roster of pediatric patients with DS in their practice who had no history of leukaemia. Potential controls with DS were randomly selected from the rosters and frequency matched to case subjects on age at leukaemia diagnosis (0, 1–3, 4–6, 7–10, 11–14, and 15–18 years). As with cases, eligible controls were required to have an available telephone in their residence and an English speaking biologic mother available for telephone interview. Permission from the child's primary care physician to contact the child's parents and written consent to interview from the mother were also required.

### Data collection

Mothers were interviewed using a structured, computer-assisted telephone questionnaire. Data on demographics, mother's medical history, pregnancy and family characteristics, and personal habits (i.e. smoking and alcohol consumption) were collected along with the index child's medical history. In addition, information regarding maternal kidney infections and UTIs occurring during the index pregnancy was collected, since medications used to treat these conditions were of interest. The child's medical history included history of mumps, chicken pox, rubella, regular measles, ear infections, colds, and bronchial infections. An open-ended question probing for any other serious illness or disease in the child was asked, and these conditions were coded according to the 9th edition of the International Classification of Diseases. The child's age at the first diagnosis of a reported infection was ascertained in years. If the child first experienced the infection before 2 years of age, then age in months at the time of infection was also collected.

Each child was assigned a reference date prior to the interview to make the data collection process similar for cases and DS controls. Cases were assigned a reference date corresponding to a date 6 months prior to leukaemia diagnosis. For controls, the reference date was a randomly selected day in the 12 months surrounding the control child's birthday in the year assigned by the frequency match. On average, interviews occurred 21 months after leukaemia diagnosis (<12 months for 59 cases, 12–24 months for 33 cases, and >24 months for 66 cases). Interviews with control mothers occurred an average of 2 months after their child's eligibility was determined.

Detailed clinical information on all DS cases was available from COG institutions. Leukaemia morphology was assigned at the COG institutions, and centrally reviewed and classified by COG investigators. Cytogenetic data were collected from the primary physician of DS controls and were centrally reviewed and verified by COG investigators.

### Statistical analyses

Odds ratios (OR) and 95% confidence intervals (CI) were used to measure the effect of maternal and child infections on leukaemia risk and were estimated using unconditional logistic regression models (SAS^©^ Version 8.02. analytical software; SAS Institute Inc., Cary, NC, USA). Analyses included all acute leukaemia combined, as well as stratified by acute leukaemia subgroups (ALL and acute myeloid leukaemia (AML)). Maternal infections during pregnancy included each type of infection, as well as infections combined into a single category and compared to those without a history of infection during pregnancy. Analyses of the association between acute leukemia and child infections were restricted to children over 1 year of age at diagnosis, or the corresponding reference date for controls, to ensure that there was an adequate window for exposure to infections. In addition, evidence indicates that leukaemia in the first year of life probably develops *in utero*, and thus postnatal exposures may not play an aetiologic role (Greaves *et al*, 2003). Six (4%) cases (one ALL and five AML) and 21 (12%) controls were younger than 1 year of age.

Children were classified as exposed if the infections occurred in the first 2 years of life and more than 1 year prior to diagnosis of leukaemia (or corresponding date for controls). The latter restriction served to minimise the possibility that infection was due to preleukaemic symptoms or conditions. Postnatal chicken pox, ear infections, and colds and bronchial infections were analysed separately along with a category for any other reported infections. The occurrences of chicken pox, ear infections, measles, colds, and bronchial infections were combined into a summary category and compared to those participants without a history of childhood infection. As the category for other infections was heterogeneous, it was not included in results reported for the summary measure of any infection but was analysed as a separate category. The effect of the number of reported infections (0, 1, 2–3) was also evaluated.

Tests for linear trend were evaluated by treating categorical variables as ordinal. Regression models were adjusted for age using a categorical variable (<2, 2–6, >6 years) to distinguish between cases occurring during the peak incidence of ALL and other childhood leukaemia cases. Due to the absence of AML cases over the age of 6, age was adjusted into two categories (<2 and ⩾2 years) for that group. Multivariate-adjusted analyses were performed to evaluate the potential confounding effects of various socio-demographic characteristics and presumed risk factors. Potential confounders identified *a priori* included the child's sex, race, birth weight, gestational age, birth order, number of older siblings, breast feeding, household income, maternal education, smoking during pregnancy, and drinking during pregnancy. Child's race was considered to be white if both parents reported being white, and non-white if either or both parents reported another race or ethnicity. A potential confounder was retained in the final multivariate model if it changed the age-adjusted parameter estimate for combined leukaemia in a meaningful way (e.g. >10%). In addition, household income was retained concurrent with maternal education to arrive at the best estimate of socioeconomic status.

## RESULTS

During the study period, a total of 210 potential cases were identified through 116 COG institutions participating in the study. Interviews were successfully completed for 158 (75%) of those eligible. Reasons for noninterview included maternal refusal (17%), physician refusal (5%), and inability to locate mother (3%). Of the successfully completed telephone interviews, 97 cases were diagnosed with ALL and 61 with AML.

Of 151 clinics contacted to provide information regarding DS controls, 47 refused or were unable to provide rosters of potential DS controls and 27 had no other age eligible children with DS to refer. In total, 77 clinics provided rosters enumerating 726 potential controls; 329 potential controls were selected by study investigators based on date of birth. Of these potential controls, a name and address was not provided to the University of Minnesota for the following reasons: the families could not be located or were no longer seen by the clinic (*N*=46), the families refused the clinic's request (*N*=19), the clinic determined the child was not eligible (*N*=18), the clinic chose not to contact them (*N*=8), or for reasons the clinics did not report (*N*=23). Names and addresses from a total of 215 mothers were forwarded to study investigators. Nine (4%) potential controls were found ineligible by the University of Minnesota. The remaining mothers of potential control children with DS were not interviewed due to refusal (11%) or inability to schedule an interview (4%). Telephone interviews were successfully completed for 173 mothers (80.5%) of the 215 potential DS controls.

Selected characteristics of the 158 cases and 173 controls are shown in Table 1

**Table 1:** Characteristics of 158 Down syndrome leukaemia cases and 173 Down syndrome controls

	**Controls**	**Childhood leukaemia cases**		**Acute lymphoblastic leukaemia cases**		**Acute myeloid leukaemia cases**	
**Characteristic or exposure**	***N*=173 (%)**	***N*=158 (%)**	***P*-value**	***N*=97 (%)**	***P*-value**	***N*=61 (%)**	***P*-value**
*Gender*							
Male	90 (52.0)	85 (53.8)	0.75	57 (58.8)	0.29	28 (45.9)	0.41
Female	83 (48.0)	73 (46.2)		40 (41.2)		33 (54.1)	
							
							
*Child's race*							
White	145 (83.8)	121 (76.6)	0.10	77 (79.4)	0.36	44 (72.1)	0.05
Non-white	28 (16.2)	37 (23.4)		20 (20.6)		17 (27.9)	
							
							
*Age at reference date (years)*							
<2	77 (44.5)	63 (39.9)	0.05	13 (13.4)	<0.0001	50 (82.0)	<0.0001
2–6	51 (29.5)	66 (41.8)		55 (56.7)		11 (18.0)	
>6	45 (26.0)	29 (18.4)		29 (29.9)		0 (0.0)	
							
							
*Birthweight (g)*							
⩽3500	136 (78.6)	124 (79.5)	0.85	80 (82.5)	0.45	44 (74.6)	0.52
>3500	37 (21.4)	32 (20.5)		17 (17.5)		15 (25.4)	
							
							
*Weeks gestation at birth*							
⩽38	85 (49.4)	69 (43.7)	0.30	45 (46.4)	0.63	24 (39.3)	0.18
>38	87 (50.6)	89 (56.3)		52 (53.6)		37 (60.7)	
							
							
*Maternal age at child's birth*							
⩽35	121 (69.9)	96 (60.8)	0.08	63 (65.0)	0.40	33 (54.1)	0.02
>35	52 (30.1)	62 (39.2)		34 (35.0)		28 (45.9)	
							
							
*Breast feeding*							
Breastfed	34 (19.7)	25 (15.8)	0.66	12 (12.4)	0.31	13 (21.3)	0.94
Bottlefed	46 (26.6)	44 (27.9)		29 (29.9)		15 (24.6)	
Both	93 (53.8)	89 (56.3)		56 (57.7)		33 (54.1)	
							
							
*Child's birth order*							
Firstborn	56 (32.4)	47 (29.8)	0.61	30 (30.9)	0.81	17 (27.9)	0.51
Not firstborn	117 (67.6)	111 (70.3)		67 (69.1)		44 (72.1)	
							
							
*Number of older siblings*							
0	56 (32.4)	47 (29.8)	0.62	30 (30.9)	0.46	17 (27.9)	0.46
1	57 (32.9)	56 (35.4)		29 (29.9)		27 (44.3)	
2	26 (15.0)	30 (19.0)		22 (22.7)		8 (13.1)	
3 or more	34 (19.7)	25 (15.8)		16 (16.5)		9 (14.8)	
							
							
*Mother's marital status*							
Married or living as married	152 (89.4)	140 (88.6)	0.74	89 (91.8)	0.71	51 (83.6)	0.13
Divorced, separated, widowed	14 (8.2)	12 (7.6)		7 (7.2)		5 (8.2)	
Never married	4 (2.4)	6 (3.8)		1 (1.0)		5 (8.2)	
							
							
*Income at child's birth*							
<$30 000	57 (33.1)	57 (36.5)	0.79	37 (38.5)	0.60	20 (33.3)	0.97
$30 000–$75 000	84 (48.9)	71 (45.5)		41 (42.7)		30 (50.0)	
>$75 000	31 (18.0)	28 (18.0)		18 (18.8)		10 (16.7)	
							
							
*Mother's educational level*							
<High school graduate	9 (5.2)	17 (10.8)	0.05	16 (16.5)	0.005	1 (1.6)	0.18
High school graduate	89 (51.8)	90 (57.0)		51 (52.6)		39 (63.9)	
>High school graduate	74 (43.0)	51 (32.2)		30 (30.9)		21 (34.5)	
							
							
*Maternal smoking during pregnancy*							
Yes	27 (15.6)	30 (19.0)	0.42	19 (19.6)	0.40	11 (18.0)	0.66
No	146 (84.4)	128 (81.0)		78 (80.4)		50 (82.0)	
							
							
*Maternal drinking during pregnancy*							
Yes	71 (41.0)	49 (31.0)	0.06	36 (37.1)	0.53	13 (21.3)	0.006
No	102 (59.0)	109 (69.0)		61 (62.9)		48 (78.7)	
							

a Numbers in tables may not sum to total number of cases/controls due to missing values.

. While the overall mean age was similar for cases and controls (5.00 and 4.64 years, respectively), cases comprised a higher percentage of the 2–6 year age group at diagnosis (*P*=0.05). Cases and controls were very similar with regard to breast feeding, birth order, number of older siblings, child's sex, birth weight, and gestational age. Study participants were predominantly white; however, cases were more likely to be non-white than controls. Mothers' mean age at the index child's birth did not differ significantly between cases and controls (32.5 years for cases and 31.9 years for controls; *P*=0.50), although case mothers were more likely to have been over the age of 35 when the index child was born (*P*=0.08). Mothers of cases, in particular mothers of ALL case patients, had an overall lower educational level than mothers of controls. However, cases and controls were very similar with respect to household income and mother's marital status. There was a tendency of mothers of control subjects to have reported more drinking during pregnancy (*P*=0.06), particularly mothers of AML cases (*P*=0.006).

An episode of shingles during pregnancy was reported for only one case mother and one control mother. No cases of rubella, regular measles, mumps, or mononucleosis during pregnancy were reported for any case or control mothers. Associations between childhood acute leukaemia and maternal UTIs during pregnancy with the index child suggested a decreased risk, although the ORs were imprecise (data not shown). The ORs for having had any UTI during pregnancy were 0.73 (95% CI: 0.38–1.42) for total leukaemia, 0.67 (95% CI: 0.28–1.59) for ALL, and 0.88 (95% CI: 0.35–2.19) for AML. No correlation was observed between report of maternal infections and report of child infections (*r*=0.01, *P*=0.83).

The results of analyses examining the association between acute leukaemia and early childhood infections are shown in Table 2

**Table 2:** Risk of acute leukaemia associated with early infections (restricted to children older than 1 year at diagnosis of leukaemia)

	**Childhood leukaemia**			**Acute lymphoblastic leukaemia**		**Acute myeloid leukaemia**	
**Infections in first 2 years of life**	**No. of controls**	**No. of cases**	**OR (95% CI)^c^**	**No. of cases**	**OR (95% CI)^c^**	**No. of cases**	**OR (95% CI)^c^,^d^**
*Any infection*^e^							
No	64	81	1.00 (reference)	43	1.00 (reference)	38	1.00 (reference)
Yes	87	69	0.55 (0.33–0.92)	52	0.52 (0.28–0.96)	17	0.59 (0.28–1.25)
							
*Frequency of infection*^e^							
0	64	81	1.00 (reference)	43	1.00 (reference)	38	1.00 (reference)
1	43	36	0.56 (0.31–1.00)	23	0.43 (0.21–0.91)	13	0.75 (0.33–1.69)
2–3	44	33	0.55 (0.30–1.02)	29	0.61 (0.30–1.22)	4	0.34 (0.10–1.13)
			*P*-Trend=0.03		*P*-Trend=0.15		*P*-Trend=0.07
							
*Chicken pox*							
No	139	142	1.00 (reference)	88	1.00 (reference)	54	1.00 (reference)
Yes	12	7	0.51 (0.19–1.37)	6	0.62 (0.21–1.81)	1	0.24 (0.03–2.16)
							
*Colds or bronchial infections*							
No	94	106	1.00 (reference)	60	1.00 (reference)	46	1.00 (reference)
Yes	57	44	0.66 (0.39–1.10)	35	0.74 (0.41–1.31)	9	0.59 (0.24–1.40)
							
*Ear infections*							
No	87	95	1.00 (reference)	53	1.00 (reference)	42	1.00 (reference)
Yes	64	53	0.73 (0.44–1.22)	42	0.77 (0.43–1.39)	11	0.62 (0.27–1.43)
							
*Other*							
No	136	145	1.00 (reference)	92	1.00 (reference)	53	1.00 (reference)
Yes	15	4	0.23 (0.07–0.73)	2	0.15 (0.03–0.75)	2	0.38 (0.08–1.90)

a Corresponding date for controls.

b Numbers in tables may not sum to total number of cases/controls due to missing values.

c ORs adjusted for age at reference date, maternal education level, and household income.

d AML adjusted for age at reference date in two categories (<2, ⩾2) due to the absence of AML cases over 6 years of age at diagnosis.

e Any infection, and frequency of infection categories include chicken pox, ear infections, measles, colds, and bronchial infections.

. Mothers of 69 cases (52 ALL and 17 AML) and 87 controls reported at least one common infection in the first 2 years of their child's life. Ear infections, colds, and bronchial infections were predominant, while chicken pox and other infections were reported less frequently. A history of measles in the first 2 years of life was reported for only two control and no case subjects. A statistically significant negative association was observed between acute leukaemia and combined report of ear infections, chicken pox, measles, colds, and bronchial infections in the first 2 years of life for combined acute leukaemia (OR=0.55; 95% CI=0.33–0.92) and ALL (OR=0.53; 95% CI=0.29–0.97). An inverse association with AML was observed that was of a similar strength but not significant (OR=0.59; 95% CI=0.28–1.25). There was evidence of a significant linear trend with increasing numbers of infection (*P* for trend=0.03); however, a dose–response relationship was not apparent in the analysis of number of infections as a categorical variable. Associations between childhood acute leukaemia and infections examined individually were consistently negative, although none reached statistical significance. A strong, significant negative association was observed between other reported infections and combined acute leukaemia (OR=0.23; 95% CI=0.07–0.73) and ALL (OR=0.15; 95% CI=0.03–0.75).

Finally, analysis for combined acute leukaemia and infection was restricted to cases (*N*=67) for whom a control from the same clinic was enrolled. Although more imprecise, there were no notable differences in the results (data not shown).

## DISCUSSION

This is the largest case–control study of leukaemia in children with DS yet conducted (Garre *et al*, 1987; Mejia-Arangure *et al*, 2003) and the first to evaluate whether the timing of common infections is associated with a reduced risk of acute leukaemia in children with DS. Infections in the first 2 years of life were associated with a reduced risk of acute leukaemia among children with DS. The strength of the association was similar across subtypes. In addition, the association between acute leukaemia and maternal infections during pregnancy, although weaker, were in the same direction as those observed for infections of the child. Thus, taken together our study offers support for the hypothesis that early exposure to common infections may be important in the aetiology of leukaemia among children with DS. Only a small proportion of children with DS will ever develop acute leukaemia, which suggests that despite increased genetic susceptibility other factors likely contribute to aetiology.

No investigation of the relationship between early infections and leukaemia in children with DS comparable to ours exists. However, a number of investigations have addressed the role of early common infections in the aetiology of leukaemia in children without DS, particularly ALL. While a few studies have observed either positive or null associations between acute leukaemia and early common infections,(Dockerty *et al*, 1999; Schuz *et al*, 1999; Naumburg *et al*, 2002), most investigations have reported negative associations(van Steensel-Moll *et al*, 1986; McKinney *et al*, 1999; Schuz *et al*, 1999; Neglia *et al*, 2000; Perrillat *et al*, 2002; Jourdan-Da Silva *et al*, 2004) Van Steensel-Moll *et al* observed a significant negative association between ALL and common colds in the first year of life (van Steensel-Moll *et al*, 1986). Neglia *et al* observed a significant trend towards reduction in the risk of ALL with increasing frequency of ear infections in the first year of life and Perrillat *et al* reported a negative association between risk of ALL and a history of four or more ear infections in the first two years of life (Neglia *et al*, 2000; Perrillat *et al*, 2002). McKinney *et al* found a significantly reduced risk of ALL with neonatal infections, specifically skin infections, and recently Jourdan-Da Silva *et al* observed a slight negative association with early infections, particularly GI infections (McKinney *et al*, 1999; Jourdan-Da Silva *et al*, 2004).

The biological explanation for a decreased risk of acute leukaemia for children with DS who experience early common infections may be very similar to that proposed for children without DS. More than 30 years ago, Sutnick *et al* proposed that the increased susceptibility to leukaemia of children with DS may be a consequence of impaired cellular immunity and found evidence of an excessive humoral immune response (Sutnick *et al*, 1971). Children with DS are highly susceptible to infection and the tendency towards an impaired immune system has been well documented. Observed defects in immune function include decreased white cell counts, impaired cell-to-cell communication, reduced antibody levels, and abnormal lymphocyte function (Fong and Brodeur, 1987; Novo *et al*, 1993). In the presence of proliferative leukocyte stress provoked by infection, inherently abnormal leukocyte stem cells in patients with DS may be at increased risk of a second mutation resulting in leukaemia (Stewart, 1961). Nevertheless, the types and frequency of infections can vary across children with DS (Selikowitz, 1992).

Greaves' hypothesis adds the importance of timing of the infection. Delayed exposure to common infections increases the pool of preleukaemic cells, while early exposure to common infections may protect children from ALL by contributing to a more normal maturation of the immune system (Greaves, 1988, 1997; Greaves and Alexander, 1993). The age-specific incidence peak between 2 and 6 years of age characteristic to ALL in the general population has been described for children with DS as well, providing epidemiological support for an environmental contributor to the aetiology of leukaemia in children with DS and supporting Greaves' hypothesis (Robison *et al*, 1984; Fong and Brodeur, 1987; Greaves, 1997). Our results, however, indicate a fairly nonspecific negative association, suggesting a possible protective effect of early infections for both ALL and AML. Genetically impaired immune systems are typically associated with lymphoblastic malignancy, although patients with DS are at increased risk of AML and ALL (Fong and Brodeur, 1987). Jourdan-Da Silva also observed a negative association with early infections that was not specific to ALL for children without DS (Jourdan-Da Silva *et al*, 2004).

It is also important to consider the changing attitudes in recent decades toward children with DS. Prior to the 1970s, children with DS were often institutionalised and deprived of all but the most basic of medical care (Van Riper and Cohen, 2001). In more recent years, children with DS have been mainstreamed at an early age with other children (often attending day care, preschool, as well being integrated into regular classrooms) (Cuckle, 1999; Van Riper and Cohen, 2001). Standards of medical care specific to DS have also been recommended (Saenz, 1999; Van Riper and Cohen, 2001). Thus, the opportunity for and recognition of early-life infections has likely increased. Unfortunately, we did not collect data on day care experience, which has been shown in some studies to be a surrogate marker for exposure to infections (Ma *et al*, 2002; Perrillat *et al*, 2002).

A relatively small sample size is a limitation of this study, resulting in some imprecise effect estimates. Response rates for study participants were similar between cases and controls. It was not possible to enroll DS controls for all primary care clinics that referred DS leukaemia cases to COG institutions, introducing potential bias if enrolled cases differ from leukaemia patients in the population of children with DS in North America. However, COG affiliated institutions treat over 90% of pediatric leukaemia patients in the United States (Ross *et al*, 1996; Liu *et al*, 2003). Therefore, it is likely that a child with DS diagnosed with leukaemia at any clinic in the United States would be referred to, and treated at a COG institution. In addition, restriction of analysis to cases with an available clinic DS control did not significantly alter our findings for combined acute leukaemia and child infections. Further, children with DS maintain a more involved clinical care regimen during childhood than children without DS, with recommended pediatrician visits for scheduled exams every 6 months to a 1 year regardless of their health (Cohen, 1999; Van Riper and Cohen, 2001). Therefore, it is unlikely that participating primary care physicians were selectively referring children with DS who were more often seen in their clinics with increased infections as potential controls.

History of infections was ascertained by maternal report, and was limited in this analysis to a set of *a priori* identified common infections. The data were collected from a structured, computer-assisted telephone questionnaire and the same standardised interview was used to collect data from mothers of cases and controls. An effort was made to collect data soon after case or control identification and use of a reference date ensured that mothers of cases and controls were recalling events over approximately the same period of time. Mothers of children with DS and leukaemia may have been motivated to actively think about their child's history, as well as their own past habits and environmental exposures. The unique use of DS controls is a strength of this study as mothers of control subjects might be similarly motivated. It is likely that any under- or over-reporting of exposure was more similar for mothers of cases and controls than would be the case in a study of children without DS. Further, it is unlikely that mothers were selectively under- or over-reporting a history of infection for themselves, but not their child, or *vice versa*, as we found no correlation between mothers reporting a history of infections for themselves and their children.

In conclusion, this study offers support for the hypothesis that early-life infections may play a protective role in the aetiology of childhood acute leukaemia in children with DS. In future investigations, molecular and biological methods may be useful in characterizing the history of infection, providing a more accurate measure of immune status, and further clarifying the relationship between an impaired immune system and leukaemia in children with DS.
